# A study of the immunomodulatory effects of coconut oil extract in broilers experimentally infected with velogenic Newcastle disease virus

**DOI:** 10.5713/ab.23.0489

**Published:** 2024-04-25

**Authors:** Muhammad Wasim Usmani, Farzana Rizvi, Muhammad Kashif Saleemi, Muhammad Zishan Ahmad, Muhammad Numan, Muhammad Zulqarnain Shakir, Nasir Mahmood, Jahanzeb Tahir

**Affiliations:** 1 Department of Veterinary Pathology, Faculty of Veterinary and Animal Sciences, Ziauddin University, Karachi, 75050, Pakistan; 2 Department of Pathology, Faculty of Veterinary Science, University of Agriculture, Faisalabad, 37000, Pakistan; 3 PMAS, ARID Agriculture University, Rawalpindi, 46300, Pakistan; 4 Veterinary Research Institute, Lahore, 54810, Pakistan; 5 Institute of Drug Discovery Technology, Ningbo University, Ningbo 315211, China

**Keywords:** Antibody Titer, Growth Performance, Immunoglobulin Y (IgY), Immunoglobulin M, Lymphoproliferative Response, Phagocytic Activity

## Abstract

**Objective:**

This study aims to evaluate the immunomodulatory effects of coconut oil extract (COE) in broilers experimentally infected with velogenic Newcastle disease virus (vNDV).

**Methods:**

A total of 150 broiler birds (day-old) were equally divided into five study groups i.e., negative control, positive control, COE-1, COE-2, and COE-3. On day 10, broilers of groups COE-1, COE-2, and COE-3 were supplemented with 1, 2, and 3 mL of COE respectively per liter of drinking water for 15 days. On day 13, 0.1 mL/bird (10^−5.25^ ELD_50_) of vNDV was inoculated in broilers of positive control, COE-1, COE-2, and COE-3 groups intramuscularly. During this study, growth performance, morbidity, and mortality rates of each study group were recorded. The antibody titer against NDV was determined on days 7, 14, 21, 28, and 35. The levels of immunoglobulins (IgY and IgM) were also determined on the 7th, 14th, and 21st days post-sheep red blood cells (SRBCs) inoculation. On day 33, avian tuberculin was injected between the 1st and 2nd toes of the left side (intradermally) to measure lymphoproliferative responses. On day 35, the phagocytic activity in the blood was assessed through a carbon clearance assay by injecting carbon black ink into the right-wing vein. The visceral organs having gross lesions were also collected for histopathology.

**Results:**

The COE significantly improved the growth performance, and lowered the morbidity and mortality rates of broilers. There was a significant rise in antibody titers against NDV and levels of IgY and IgM antibodies against SRBC in COE-supplemented broilers. The lymphoproliferative response and phagocytic activity were also enhanced. Among COE-supplemented groups, the broilers of the COE-3 group showed a significant increase in growth performance and boosted immune defense.

**Conclusion:**

Coconut oil extract has the potential to boost the growth performance and immune status of broilers. It can be used effectively as a feed additive and alternative to antibiotics to prevent the spread of infectious poultry pathogens.

## INTRODUCTION

Newcastle disease (ND) is a viral disease that is caused by the Newcastle disease virus (NDV). It is a negative sense RNA virus which belongs to the *Paramyxoviridae* family [1]. It is the second most prevalent disease in birds, reported in about 56 of the 167 member countries of the World Organization of Animal Health (WOAH) [2]. Clinically, ND develops respiratory distress, nervous signs such as tremors and paralysis, diarrhea, decreased egg production, and high mortality rates in poultry birds [3]. However, disease severity can vary, depending on strain virulence and the immune status of the affected host [4].

The ND virus is categorized into three serotypes i.e., lentogenic, mesogenic, and velogenic. The velogenic strain produces serious neurological and respiratory problems in poultry birds [5]. Clinical signs may range from 100% mortality among non-vaccinated birds to a drop in egg production among seemingly healthy, well-vaccinated layers. Grossly, congestion and hemorrhage are prominent, particularly in the trachea, proventriculus, cecal tonsils, and intestine of the birds [6]. Those birds that are infected with the neurotropic (velogenic Newcastle disease virus [vNDV]) strain remain vigilant before neurological signs development such as ataxia, torticollis, unilateral leg or wing paralysis without showing any gross lesions [7]. In layer birds, multiple NDV vaccines play a substantial role in the development of persistent immunity and they do not show signs of infection except a drop in egg production [8].

In the recent past, various types of vaccines have provided protection against NDV as well as other viral infections and control diseases spread effectively [9]. However, some strains of NDV can undergo genetic changes over time, potentially leading to the emergence of new virulent strains. In addition to this, various control strategies such as strict biosecurity and efficient flock management have been adapted to overcome disease load but outbreaks in the field are still the main concern [10]. Nowadays, the research and development sectors of various countries are addressing effective ways to control the emergence and spread of infectious diseases by exploring natural alternatives such as organic oils and dietary supplements [11].

Coconut oil, a naturally occurring substance is a mixture of multiple active ingredients that enhance the growth performance and immune status of animals and birds, ultimately reducing the disease load and impact in the livestock and poultry sectors [12]. One such active ingredient is lauric acid (LA), a medium-chain fatty acid (MCFA), that has been studied for its potential immune-modulating effects against viral and bacterial infections [13]. It has been shown to possess antiviral properties against several enveloped viruses that are lipid-coated such as influenza virus, paramyxovirus, leukemia virus, and pneumono virus [14] by disrupting their membrane, interfering with virus assembly and maturation [15]. It acts by solubilizing the lipids and phospholipids in the envelope of the virus, causing the disintegration of the virus envelope [16].

However, the specific role of COE in modulating the immune response against NDV in poultry is not well-documented in scientific literature. Therefore, this study aims to evaluate the potential role of coconut oil extract (COE) in the growth performance and immune status of broiler birds against Newcastle disease virus (NDV).

## MATERIALS AND METHODS

### Ethical statement

This experimental study was carried out in compliance with the Institutional Bioethics Committee (IBC), Ref. No. 7145; dated: 15-10-2021, University of Agriculture, Faisalabad, Pakistan.

### Newcastle disease virus procurement

A previously identified and confirmed NDV by reverse transcriptase polymerase chain reaction (RT-PCR) [17] was propagated by injectjion into the allantoic cavity of 9-day-old embryonated eggs followed by incubation at 37°C for the next 7 days. The mortality of embryos was recorded to identify the velogenic strain of NVD. The embryo lethal dose (ELD_50_) of NDV was calculated by Reed and Munch method as described by Villegas and Purchase (1989). The allantoic fluid of each embryonated egg was tested for NDV titer by hemagglutination (HA) test [18].

### Preparation of coconut oil extract

The COE was prepared by the soxhlet extraction method i.e., heated in water-bath with 10M solution of sodium hydroxide (NaOH). This converted the coconut oil into two distinct layers of LA and glycerin followed by the separation of LA as a COE to prepare 5% (w/v) suspension [19].

### Experimental study

A total of 150, day old broiler chicks were equally divided into 5 study groups i.e., negative control, positive control, COE-1, COE-2, and COE-3, and kept in an experimental room at the University of Agriculture, Faisalabad for 35 days. On day 10, the broilers in COE-1, COE-2, and COE-3 groups were supplemented with 1, 2, and 3 mL of COE per liter of drinking water respectively. On day 13, the broilers of positive control, COE-1, COE-2, and COE-3 groups were experimentally infected with 0.1 mL/bird (10^−5.25^ ELD_50_) of vNDV intramuscularly [18]. Clinical signs, morbidity, and mortality rate (%) were recorded daily while growth performance i.e., body weight gain (grams) was calculated every week.

### Scoring of clinical signs

The clinical signs observed in the broilers of the study groups were scored according to (OIE 2021, Manual of Diagnostic Tests and Vaccines for Terrestrial Animals) with some modifications such as; 0 = no respiratory signs, no greenish diarrhea, no opisthotonos, no torticollis, no conjunctivitis; 1 = respiratory signs, no greenish diarrhea, no opisthotonos, no torticollis, no conjunctivitis; 2 = respiratory signs, greenish diarrhea, no opisthotonos, no torticollis, no conjunctivitis; 3 = respiratory signs, greenish diarrhea, opisthotonos, no torticollis, no conjunctivitis; 4 = respiratory signs, greenish diarrhea, opisthotonos, torticollis, no conjunctivitis; 5 = respiratory signs, greenish diarrhea, opisthotonos, torticollis, conjunctivitis.

### Determination of antibody titer against Newcastle disease virus

On days 7, 14, 21, 28, and 35 of the experiment, the blood (without ethylenediaminetetraacetic acid) was collected from the wing vein of broilers, and serum was separated to check the levels of antibodies against NDV via hemagglutination and inhibition (HA/HI) test [20].

### Determination of IgY and IgM levels against SRBCs

On the 7th, 14th, and 21st days-post inoculation of 1% SRBCs, blood was collected followed by serum separation, inactivation by heat at 56°C for 30 min and analyzed for mercaptoethanol (ME) sensitive (immunoglobulin Y [IgM]), and ME resistant (IgY) anti-SRBC antibodies [20]. Briefly, 50 μL of serum was added in an equal amount of phosphate-buffered saline (PBS) in the first column of a 96-well plate and incubated for 30 min at 37°C. A two-fold serial dilution of each serum was made followed by the addition of 50 μL of 1% SRBC to each well and incubated for 30 min at 37°C for the determination of total protein level. For IgY response, a volume of 50 μL of 0.01 M ME was added in serum instead of PBS. The difference between total protein and IgY response was equal to IgM level.

### Determination of lymphoproliferative response against avian tuberculin

On day 33, 0.2 mL/bird of avian tuberculin was injected between the 1st and 2nd left toes of 7 broilers (intradermally) from each study group. On the contrary, 0.2 mL/bird of normal saline (NS) was injected between 1st and 2nd right toes of the same broiler birds as a control [20]. An increase in cutaneous thickness (mm) was measured with the help of screw gauge pre-(zero reading) and post-avian tuberculin administration i.e., 24, 48, and 72 hours.

### Determination of phagocytic index

The phagocytic ability of chicken circulatory macrophages was determined on day 35 via carbon clearance assay (CCA). Briefly, the black Indiana ink (Pelikan, 4001) was centrifuged at 3,000 rpm for 30 min and the supernatant fraction was collected followed by injecting into the right-wing vein of the 7 broilers (1 mL/kg/bird) from each study group. Blood samples of 2 mL/chick were collected from the left-wing vein of the same broilers at 0 min (pre-), 3, and 15 min of post-injection. These blood samples were immediately transferred into 4 mL of 1% sodium citrate, centrifuged at 1,000 rpm for 4 min and the supernatant’s optical density (OD) was measured at 640 nm wavelength. The relative amount of un-phagocytized carbon particles remaining in the supernatant of each bird was determined by subtracting the OD value at 3 and 15 min from that of 0 min [20].

### Histopathological examination

During and at the end of the experiment, histopathological changes associated with NDV infection were determined by collecting visceral organs i.e., trachea, proventriculus, and intestine having gross lesions. These tissue samples were preserved in 10% neutral buffer formalin and processed by paraffin embedding technique to find pathological alterations [21]. Tissue blocks were cut at 5 μm thickness and mounted on glass slides and then stained with hematoxylin and eosin (H&E). These stained glass slides were examined under the microscope to study degenerative cellular changes in these tissue samples.

### Statistical analysis

The data thus obtained were subjected to one-way analysis of variance and group means were compared through Tukey’s test at 95% confidence interval (CI) with SPSS statistical software (IBM SPSS Statistics, Armonk, NY, USA). The p-values less than 0.05 (p<0.05) were considered significant.

## RESULTS

The ELD_50_ was calculated as 10^−5.25^/0.1 mL of vNDV and it showed a high geometric mean titer i.e., 1:512 by hemagglutination (HA) test.

### Clinical signs and gross lesions

**Figure 1:**
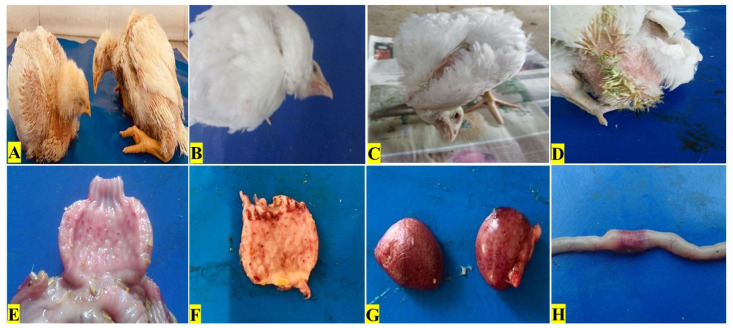
Clinical signs and gross lesions in broilers experimentally infected with velogenic Newcastle disease virus.

Clinical signs i.e., ruffled feathers, difficulty in breathing, torticollis, nervous signs, and greenish diarrhea were prominent in the broilers of the positive control group (Figure 1A–1D). On the other hand, the COE-supplemented broilers showed fewer clinical signs against NDV lethal effects. The negative control group scored 0 while the positive control scored 5 as shown in Table 1. Grossly, in NDV-infected broilers, mild to severe hemorrhages were prominent on the mucosal surface of the proventriculus (Figure 1E and 1F). Splenomegaly and intestinal ulcers were also seen in NDV-infected broilers (Figure 1G and 1H).

### Growth performance

**Figure 2:**
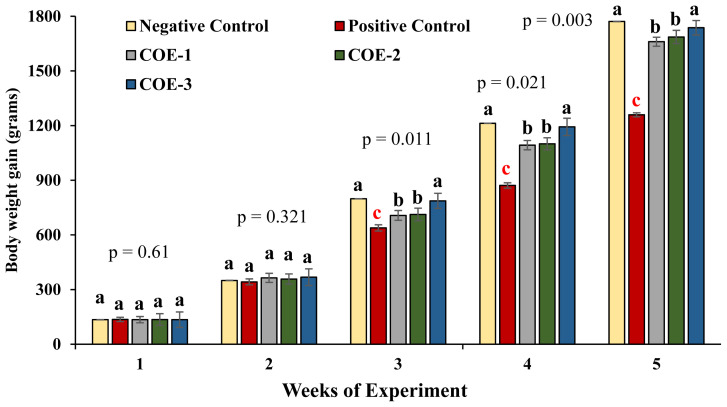
Comparison of growth performance of broilers experimentally infected with velogenic Newcastle disease virus and supplemented with COE. COE, coconut oil extract. ^a–c^ Significant at p<0.05.

During the 3rd to 5th weeks, the body weight of broiler birds of COE-supplemented groups was significantly higher as compared to positive control (p<0.05). Among COE-supplemented groups, the COE-3 group showed significantly higher body weight i.e., 787.1±64.9, 1,193±257.8 and 1,737.1±305.9 during 3rd, 4th, and 5th weeks respectively from COE-1 and COE-2 groups (p<0.05). However, a non-significant difference in body weight was observed among COE-1 and COE-2 groups (p>0.05) as shown in Figure 2.

### Morbidity and mortality rateAs

**Figure 3:**
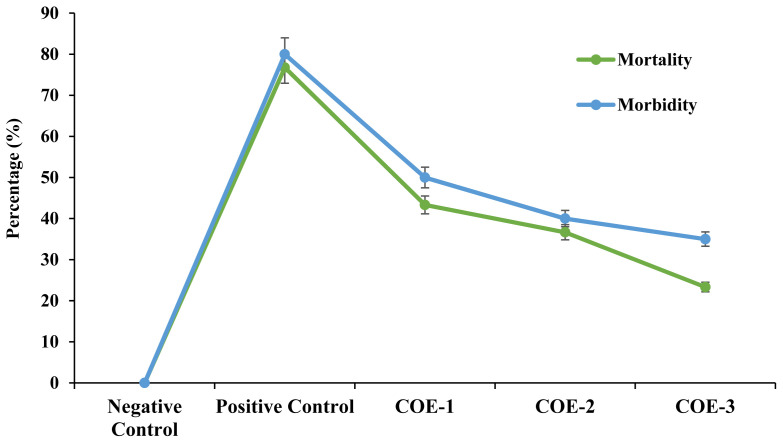
Morbidity and mortality rates of broilers experimentally infected with velogenic Newcastle disease virus and supplemented with COE. COE, coconut oil extract.

The broilers of the positive control group showed significantly higher morbidity and mortality i.e., 76.77% and 80% respectively as compared to the rest of the study groups throughout the experimental trial (p<0.05). However, a non-significant difference in morbidity (43.33%, 36.67%, and 23.33%) and mortality (50%, 40%, and 35%) was observed among COE-1, COE-2, and COE-3 groups (p>0.05) as shown in Figure 3.

### Antibody titer against Newcastle disease virus

**Figure 4:**
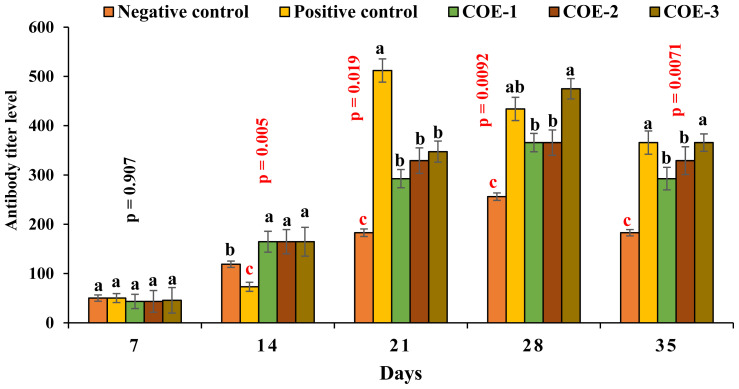
Antibody titer (Geometric Mean Titer) against NDV of broilers experimentally infected with vNDV and supplemented with coconut oil extract (COE). vNDV, velogenic Newcastle disease virus; COE, coconut oil extract. ^a–c^ Significant at p<0.05.

The antibody titer of COE-supplemented broilers was significantly higher as compared to control groups which showed less antibody titer (below the protective level) against NDV on day 14. However, on day 21, the positive control broilers showed significantly higher antibody titer i.e., 512±56 as compared to COE-1 (292.6±48.9), COE-2 (329.1±49.2), and COE-3 (347.4±60.5) groups (p<0.05). On days 28 and 35, the COE-3 group showed significantly higher antibody titers i.e., 475±74.5 and 365.7±46.8 from COE-1 (365.7±68.3 and 292.6±56.8) COE-2 (365.7±68.1 and 329.1±54.9) groups (p<0.05) as shown in Figure 4.

### Response of IgY and IgM antibodies against sheep RBCs

**Figure 5:**
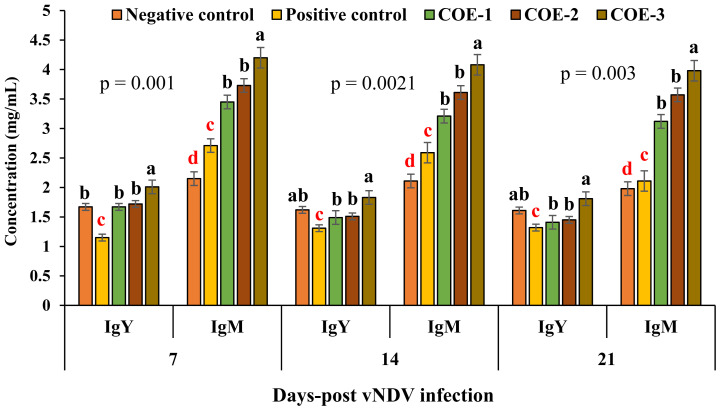
Concentration of IgY and IgM in blood serum of broilers experimentally infected with vNDV and supplemented with coconut oil extract. vNDV, velogenic Newcastle disease virus; Ig, immunoglobulin; COE, coconut oil extract. ^a–d^ Significant at p<0.05.

Post inoculation of sheep RBCs, the concentrations of IgY and IgM antibodies in COE-supplemented broilers (COE-1, COE-2, and COE-3) were significantly different from positive control broilers (p<0.05) as shown in Figure 5. On days 7, 14 and 21, COE-3 group showed the highest response of IgY (2.01±0.2, 1.83±0.2, and 1.81±0.2) and IgM (4.20±0.3, 4.08±0.3, and 3.98±0.3) as compared to the positive control (IgY = 1.15±0.1, 1.31±0.1, and 1.32±0.1; IgM = 2.71±0.2, 2.59±0.3, and 2.11±0.3), COE-1 (IgY = 1.67±0.1, 1.49±0.2, and 1.41±0.2; IgM = 3.45±0.2, 3.21±0.2, and 3.12±0.2), and COE-2 (IgY = 1.72±0.1, 1.51±0.1, and 1.45±0.1; IgM = 3.73± 0.2, 3.61±0.2, and 3.57±0.2) groups.

### Lymphoproliferative response against avian tuberculin

**Figure 6:**
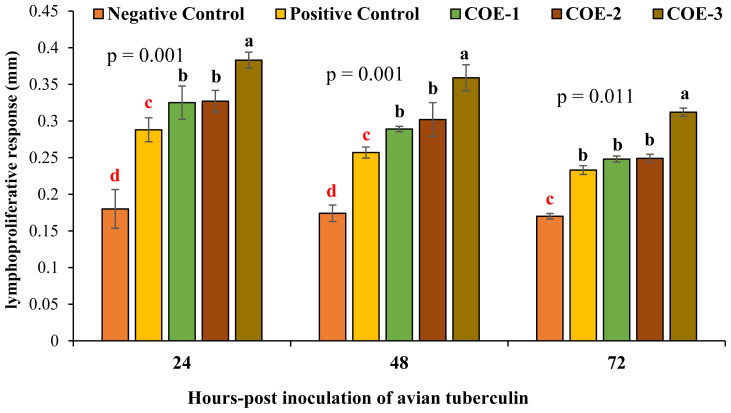
Determination of lymphoproliferative response (mm) of broilers experimentally infected with vNDV and supplemented with COE. vNDV, velogenic Newcastle disease virus; COE, coconut oil extract. ^a–d^ Significant at p<0.05.

Post 24, 48, and 72 hours of inoculation of avian tuberculin, the COE-supplemented broilers showed a significantly higher lymphoproliferative response (cutaneous thickness, mm) as compared to control groups (p<0.05) as shown in Figure 6. The COE-3 group broilers showed maximum lymphoproliferative response i.e., 0.383±0.11, 0.359±0.17, and 0.312±0.15 while negative control broilers showed least response i.e., 0.180±0.07, 0.174±0.09, and 0.170±0.10.

### Phagocytic index

**Figure 7:**
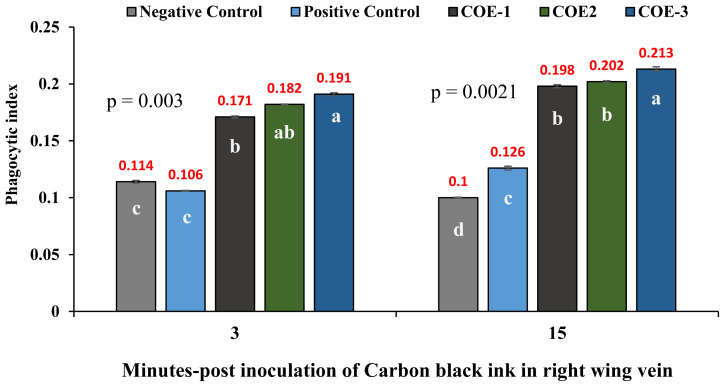
Determination of phagocytic index of broilers experimentally infected with vNDV and supplemented with COE by CCA. vNDV, velogenic Newcastle disease virus; COE, coconut oil extract; CCA, carbon clearance assay.

Post inoculation of carbon black ink, the macrophages activity of COE-supplemented broilers was significantly higher at 3 and 15 min as compared to positive control positive broilers (p<0.05) as shown in Figure 7. The COE-3 group showed significant higher i.e., 0.191±0.003 and 0.213±0.005 while positive control showed the least i.e., 0.106±0.001 and 0.126 ±0.004 phagocytic index.

### Histopathological examination

**Figure 8:**
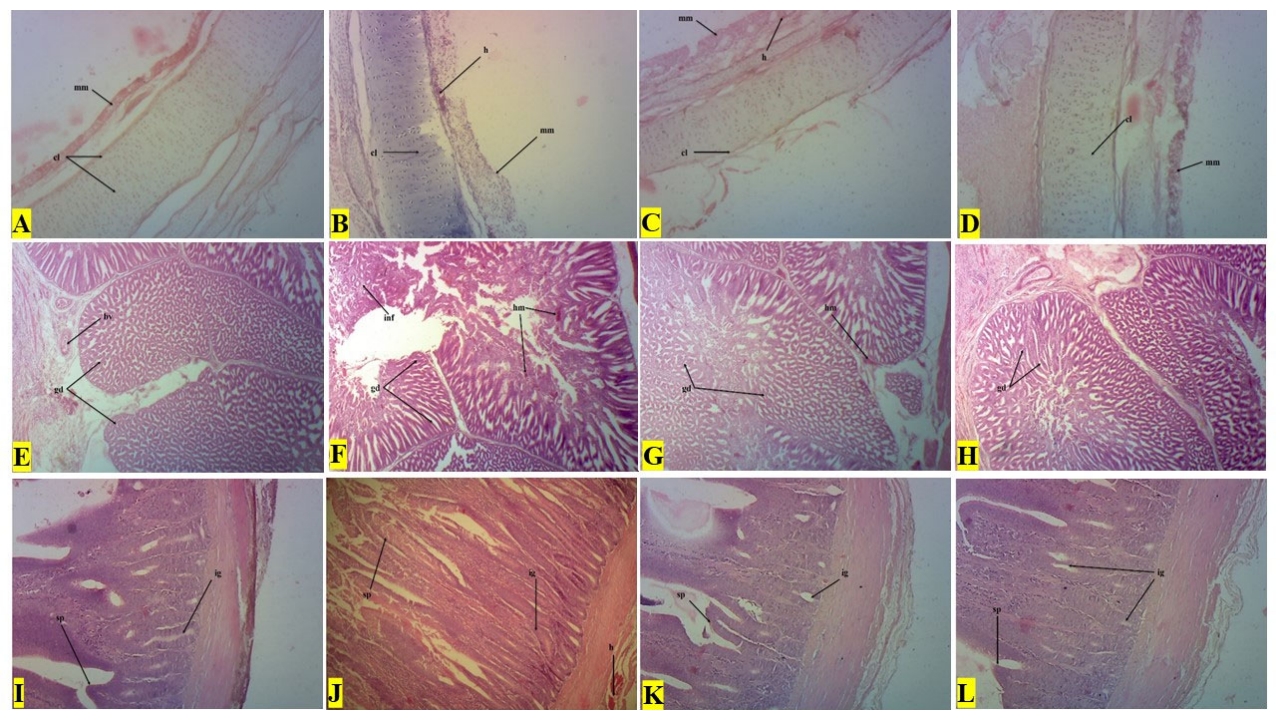
Histopathological micrograph of the trachea (first row), proventriculus (second row), and intestines (third row) of broilers experimentally infected with velogenic Newcastle disease virus and supplemented with coconut oil extract.

The histopathological textures of visceral organs i.e., trachea, proventriculus, and intestine are described in Figure 8. The negative control broilers showed no lesions in the trachea (Figure 8A), proventriculus (Figure 8E), and intestine (Figure 8I). Opposite to this, positive control broilers showed severe degenerative changes such as epithelial hyperplasia, lymphocytes infiltration, and hemorrhages (h) on tracheal cartilage (cl) surface (Figure 8B). The atrophy and hemorrhages (h) in the lamina propria of tubular glands (gd), and inflammatory (inf) cell infiltration in the proventriculus were more prominent (Figure 8F). The intestine showed necrotic surface epithelium (sp) with hemorrhages (h) and severely altered intestinal glands (ig) (Figure 8J). However, the COE-supplemented broilers showed normal cartilage (cl) and less prominent hemorrhages on mucosal epithelial (mm) (Figure 8C and 8D), fewer hemorrhages with Normal tubular glands (gd) in the proventriculus (Figure 8G and 8H), mild to moderate cellular changes i.e., Enterocytes and lymphocytes infiltration in the intestinal texture (Figure 8K and 8L).

## DISCUSSION

Nowadays, ND outbreaks and prevention are major concerns worldwide. Due to growing concerns regarding ND-associated production losses despite vaccination, this research was focused on evaluating the naturally occurring substances to enhance protection and production potential [17]. In the present study, COE supplementation significantly improved the growth performance of broilers indicating its potential as a performance enhancer. Consistent with previous studies [3,21] our results revealed the advantages of COE in improving the growth performance. This has been attributed to improve intestinal health and maintain the integrity of the intestinal mucosal wall.

The major products of COE are short-chain fatty acids mainly MCFAs such as LA. The MCFAs supplementation in broiler feed improved body weight and reduced morbidity and mortality in broilers. It is believed that LA has antimicrobial properties that can help in controlling the growth of certain bacterial pathogens in the gastrointestinal tract, leading to improved gut health. A healthy gut environment improves nutrient utilization, which in turn contributes to better growth performance in birds [22,23].

Coconut oil extract supplementation improved the body weight of broilers irrespective of its inclusion level, but a higher increase in body weight was observed when the highest level of the extract was used indicating a wide safety margin [24]. Lauric acid, a major product of coconut oil extraction has been studied for its potential effect as a growth promoter in broilers. During these studies, dietic inclusion of LA had a positive impact on growth performance and feed efficiency [16]. During the current study, the morbidity and mortality rates were significantly lower in COE-supplemented broilers as compared to vNDV-affected broilers. A number of effects have been reported in the literature with respect to LA supplementation in the broiler diet [25].

The anti-inflammatory properties of LA appeared to have been associated with varying protection against viral infections. Improved nutrient digestibility and absorption following LA inclusion may be responsible for weight gain and less mortality. The antimicrobial properties of LA have been described in earlier studies [11,26,27]. This property of LA may also have been contributing to the improved feed efficiency in broilers. A very limited number of studies have addressed the effects of LA supplementation on the immune response of modern poultry [28,29]. During the present study, the antibody titers against NDV and SRBCs were higher in the COE-supplemented groups when compared to the negative control group (broilers without COE-supplementation). Birds exposed to vNDV and supplemented with COE extract had higher titers when compared to vNDV-exposed birds without extract supplementation. However, extensive studies on the effects of LA supplementation on the immune response of broilers are very limited.

Lauric acid supplementation upregulated all types of immunoglobulins (IgA, IgM, and IgY) and downregulated all inflammatory cytokines [25]. In another study, monolaurin improved the protection against infectious bronchitis following its supplementation in diet [30]. It is also reported that high antibody titers against Newcastle disease virus when supplementated with α-monolaurin [31]. Similar findings have reported that birds fed on organic acids-based diets have heavier bursa of Fabricius and thymus. One can conclude from this and earlier studies, that dietary supplementation with LA improves immune response against various poultry pathogens and also improves the weight of lymphoid organs [32,33]. Similar results were noted in elevated humoral immunity against SRBCs [20,28] as an outcome of high IgM production. Previously, it has been reported that LA reduces inflammatory responses by reducing the effects on total leucocytes, mono- and poly-morphonuclear circulating cells [29], c-reactive protein, and nitric oxide, as well as the number of white blood cells and levels of tumor necrosis factor (TNF-) and interleukin (IL)-6 in adipose tissue [30,31]. A time-dependent increase in HI titer in chicken serum vaccinated with killed ND vaccine via intramuscular following ginseng stem leaf saponins supplementation has also been reported [32,33].

The complex immune system of birds is made up of several cellular and soluble components that must cooperate to generate a protective immune response [34]. Raised levels of humoral and cellular immune responses were reported in broiler chicks by indirect enzyme-linked immunosorbent assay when immunized with *Momordica cocshinchinensis* extract along with the ND vaccine [35]. Similar findings were published where high levels of serum antibodies i.e., IgY and IgM were found protective against NDV [36,37]. This supplementation enhances immunological performance which in turn improves humoral immunity [38,39]. Using globulin concentration as an indication, one can gauge an immune response. As a result of peripheral T4-T3 conversion and hyperthyroidism produced by organic acid feeding, birds had improved immunocompetence and bursa expansion, according to the data [40]. It suggests that the production of T-cells, B-cells, and phagocytes plays a major role in cell-mediated immunity. It is important to note that, there is a time-related shift in T-cells and intradermal administration of avian tuberculin stimulates T-cells which surges chemokine/cytokines and results in increased skin thickness [20].

## CONCLUSION

The present study suggests that the inclusion of the extract of coconut oil in the feed of broilers improves body weight gain and immunity. Immune response parameters were also evident. The exact mechanism(s) behind these effects need further investigation. It can be used to replace antibiotics to overcome antimicrobial resistance which is a major public health and food safety concern nowadays in the poultry industry. Furthermore, it is important to note that, during this study three different dose levels i.e., 1, 2, and 3 mL per liter of drinking water were applied and 3 mL/L showed better results as compared to the rest of the dose levels in broiler birds. Therefore, there is a need for hours to optimize the dosage of LA for operative supplementation and to fully understand the mechanism of action to control the spread of infectious diseases effectively.

## Figures and Tables

**Table 1:** Clinical signs scoring of broilers experimentally infected with velogenic Newcastle disease virus and supplemented with coconut oil extract

Study groups	Clinical signs					Score

	Respiratory signs	Greenish diarrhea	Opisthotonos	Torticollis	Conjunctivitis	
Negative control	−	−	−	−	−	0
Positive control	+	+	+	+	+	5
COE-1	+	+	+	+	−	4
COE-2	+	+	+	−	−	3
COE-3	+	+	−	−	−	2

COE, coconut oil extract.
